# Milk from Halari Donkey Breed: Nutritional Analysis, Vitamins, Minerals, and Amino Acids Profiling

**DOI:** 10.3390/foods12040853

**Published:** 2023-02-16

**Authors:** Renu Garhwal, Anuradha Bhardwaj, Karnam Sangwan, Rahul Mehra, Yash Pal, Varij Nayan, Mir Asif Iquebal, Sarika Jaiswal, Harish Kumar

**Affiliations:** 1 Amity Institute of Biotechnology, Amity University Rajasthan, Jaipur 303002, India; 2 Indian Council of Agricultural Research—National Research Centre on Equines, Hisar 125001, India; 3 Food Science & Technology MMICT & BM(HM), Maharishi Markandeshwar (Deemed to Be University), Mullana, Ambala 133203, India; 4 Indian Council of Agricultural Research—Central Institute for Research on Buffaloes, Hisar 125001, India; 5 Division of Agricultural Bioinformatics, Indian Council of Agricultural Research-Indian Agricultural Statistics Research Institute, New Delhi 110012, India

**Keywords:** donkey milk, nutritional composition, minerals, vitamins, amino acids

## Abstract

This current research set out to characterize Halari donkey milk by investigating its nutritional constituents, including its proximate analysis, water activity, titratable acidity, energy, and microbiological analysis. A comprehensive profiling of vitamins, minerals, and amino acids was also carried out. It was found that the composition of Halari donkey milk was consistent with previously published donkey milk literature and was comparable to that of human milk. Halari donkey milk has low 0.86 ± 0.04% fat content, 2.03 ± 0.03% protein content, 0.51 ± 0.05% ash content, and high 5.75 ± 0.15% lactose content making it sweet and palatable. The energy content of Halari donkey milk was 40.39 ± 0.31 kcal/100 g, and the water activity ranged from 0.973 to 0.975. Titratable acidity was 0.03 ± 0.01%. Halari donkey milk can be considered acceptable and microbiologically safe, having low total plate count and yeast and mould counts. Mineral testing revealed that Halari donkey milk included significant amounts of magnesium, sodium, calcium, potassium, phosphorus, and zinc. The concentration of different vitamins and amino acids such as isoleucine and valine also contribute to the nutritional value of Halari donkey milk.

## 1. Introduction

Each mammal’s milk is uniquely designed to meet the infant’s nutritional needs and give passive perinatal immunization. The ideal infant diet is unquestionably human breast milk, at least during the first four months of life [1]. There are a few situations, such as lack of milk ejection, unwell mothers, or orphans, when it may be required to find a suitable substitute. Due to the amount of lacteal secretions necessary to meet the demand for milk and other dairy products for human nutrition, cows are the most common species of dairy animal in the world [2]. However, cow milk is unsuitable for newborns suffering from cow milk protein allergy (CMPA) [3]. Milk from other animal species, including goats, horses, and donkeys, could be used as human milk substitutes. Worldwide interest in donkey milk as a substitute for human milk has grown over the past ten years. Due to its unique qualities, donkey milk appears to be a remarkable replacement to solve the problem for those who are intolerant to heavily hydrolysed milk-based formulas and for children with CMPA [4]. Donkey milk exhibits organoleptic characteristics and a chemical composition comparable to human milk [5]. In terms of its high lactose and low protein content and even lower level of fat percentage, donkey milk is most comparable to human milk [6,7]. Donkey milk has a lower amount of somatic cells (4.0–4.87 log cells/mL), low microbial level (3.10–4.09 log Cfu/mL), and a total bacterial count of 3.69 log Cfu/mL, which contribute to an extended shelf life and consistent quality [7].

There is much literature on studies related to milk composition in different donkey breeds worldwide. These include the Sardinian donkey [8], Amiata donkey [9], Jiangyue donkeys [5], Martina Franca jennies [10], Ragusano jennets [11,12], Indian small grey breed [13], domestic Balkan donkey breed [14], and Arcadian donkey [7]. Compared to the milk of the major dairying animals such as cow, buffalo, goat, camel, and sheep, donkey milk has a significantly different composition. It has a higher proportion of lactose and less fat and protein content than bovine milk [15]. The low protein content in donkey milk offers real benefits because a high protein intake in formula-fed infants is mostly associated with the cases of early adiposity rebound [16]. 

In India, there are three registered breeds of donkey: Spiti, Halari, and Kachchhi [17]. This research is focused on the compositional and nutritional analyses of Halari donkey milk. There is no literature relating to this breed’s milk composition and the breed’s milk has not yet been classified. The Halari donkey breed is a recently registered one (Accession No: INDIA_DONKEY_0400_HALARI_05002) that is primarily found in the state of Gujarat, India. They may migrate across great distances and have a predominance of white colouring (National Bureau of Animal Genetic Resources, Karnal). This breed is primarily found in the Gujarat’s Jamnagar, Dwarika, and Rajkot districts of India. It is the most prevalent breed of local donkeys. They are primarily utilized for material transportation as pack animals [18]. This present work aims to examine the nutritional, microbial analysis of Halari donkey milk, along with detailed profiling of minerals, vitamins, and amino acids using various analytical, biochemical, and microbiological approaches.

## 2. Materials and Methods

### 2.1. Sample Collection 

Milk samples were collected from healthy Halari jennies, reared at a donkey farm at the ICAR-National research centre on equines, Hisar, Haryana, India. Eighty-one raw samples of Halari donkey milk from nine different animals were sorted in sterile HDPE bottles (Tarsons Products Pvt. Ltd., Kolkata, India) between the 21st and 30th week of lactation (during the months of August to October). The sampling was carried out in compliance with the Institutional Animal Ethics Committee’s rules and regulations (IAEC). Sampling was done through manual milking, twice a day (morning and evening) under aseptic conditions, completely evacuating the milk. The samples obtained during both shifts (morning and evening) were pooled. The pooled samples from each animal were then stored at −20 °C for further analysis.

### 2.2. Nutritional Analysis of Halari Donkey Milk

Nutritional analysis of donkey milk was performed in terms of its total solids, solids not fat (SNF), and casein content according to IS 1479 (P-2): 1961. Lactose content was determined, as previously mentioned [19,20]. Fat percentage was determined by Gerber method, ash content by muffle furnace method, and protein content by Kjeldahl’s method, and titratable acidity was measured following the method [21]. Total carbohydrates were calculated by difference method [22] as per equation:

$$Carbohydrates\left(\%\right)=100-\left(moisture\;\%+fat\;\%+protein\;\%+ash\;\%\right)$$

Water activity of donkey milk was measured by water activity meter (Novasina AG, Lachen, Switzerland). Gross energy was calculated with the coefficient values of 9.11 for fat, 3.95 for lactose, and 5.86 for protein. Microbiological analysis of Halari donkey milk was performed in an aseptic atmosphere to avoid any cross contamination. The total plate count (TPC) and total yeast and mould count in milk samples were assessed using method no. Indian standards IS 5402 (2012) and IS 5403, (1999), respectively.

### 2.3. Minerals, Vitamins, and Amino Acids Profiling of Halari DM

Halari donkey milk was evaluated for its mineral, vitamin, and amino acid profile. Mineral profiling samples were prepared by PerkinElmer’s TITAN MPS microwave digestion system and analysed by NexION 2000 ICP-MS (PerkinElmer Inc., Shelton, CT, USA) by the Interstellar testing centre Pvt. Ltd., Panchkula, India [23]. Vitamins in donkey milk were analysed by the FICCI research and analysis centre (FRAC) following the protocol of Haddadin et al. (2008) [24]. Amino acid profiling was examined by the Interstellar testing centre Pvt. Ltd., Panchkula, India, using high-performance liquid chromatography (HLPC) by hydrolysis of milk samples with hydrochloric acid following the method of Guo et al. (2007) [5].

### 2.4. Statistical Analysis

All the observations used in the analysis indicated above were shown as mean ± SD using the 2019 version of Microsoft Excel (Microsoft Corporation, One Microsoft Way, Redmond, WA, USA). 

## 3. Results

### 3.1. Nutritional Analysis of Halari Donkey Milk

Table 1 lists the descriptive statistics of physicochemical characteristics of Halari donkey milk. Gross composition of Halari donkey milk was characterized by very low fat content (0.86 ± 0.04%) and high amounts of lactose (5.75 ± 0.15%). Total solids and solids not fat (SNF) accounted for 9.61 ± 0.08% and 8.81 ± 0.14%, respectively. Low protein content (2.03 ± 0.03%) was recorded in Halari donkey milk, with fair amounts of carbohydrate content (6.23 ± 0.19%). The descriptive statistics of mineral content in Halari donkey milk is represented in Table 2. The mineral content calculated in the form of ash percentage was 0.51 ± 0.05%. Titratable acidity (TA) is a metric which is useful for characterising the buffering capacity and natural acidity as it measures the overall dissociated and undissociated acid content. The TA in Halari donkey milk was recorded as 0.03 ± 0.01%. Donkey milk has a higher water activity, which is dependent on a complex biochemical makeup of the milk samples. Water activity (a_w_) in the Halari donkey was depicted to be 0.973–0.975. A low total plate count (TPC) of 3.5–3.9 × 10^4^ cfu/mL and a yeast and mould count of 2.9–3.2 × 10^3^ cfu/mL were recorded in milk samples. Due to its natural antibacterial action, donkey milk has a significantly low microbial count when compared to milk from other dairy animals [25]. 

### 3.2. Minerals, Vitamins, and Amino Acids Profiling of Halari DM

Overall, mineral concentration is presented in Table 2. Major minerals present in Halari donkey milk were magnesium (Mg), sodium (Na), calcium (Ca), Potassium (K), Phosphorus (P), and zinc (Zn). Among all the detected minerals, Ca was most abundant, having a concentration of (61.25 ± 0.93 mg/100 mL). The concentration of other minerals observed in Halari donkey milk were potassium (47.39 ± 0.69 mg/100 mL), sodium (13.84 ± 0.92 mg/100 mL), phosphorus (32.99 ± 0.76 mg/100 mL), and zinc (1.83 ± 0.05 mg/100 mL), respectively. The average concentration of vitamins in Halari donkey milk are presented in Table 3. Among the vitamins, thiamine, riboflavin, niacin, pantothenic acid, pyridoxine, and biotin were major vitamins detected in Halari donkey milk. Vitamin B5 (pantothenic acid) (0.206 ± 0.2 mg/100 mL) was present in highest concentration among all vitamins, followed by B3 niacin (0.135 ± 0.02), B1 thiamine (0.023 ± 0.01 mg/100 mL), B6 pyridoxin (0.010 ± 0.02 mg/100 mL), B2 riboflavin (0.006 ± 0.001 mg/100 mL), and B7 biotin (6.808 ± 0.39 mcg/100 mL). 

Concentration of amino acids in Halari donkey milk are presented in Table 4. Isoleucine was observed to have the highest concentration among essential amino acids followed by valine. Phenylalanine was present in the lowest concentration. The body’s physiological processes including immunity, growth, fatty acid metabolism, protein metabolism, and glucose transfer are all impacted by isoleucine. Other essential amino acids such as leucine, lysine, methionine, and phenylalanine were also detected in Halari donkey milk. Among all the non-essential amino acids detected in Halari donkey milk, proline was present in highest concentration followed by arginine, serine, glutamic acid, histidine, glycine, alanine, and aspartic acid, Table 4. Proline plays a vital role in protein synthesis, being a crucial part of protein structure, and is also involved in the metabolic process as the synthesis of arginine, polyamines, and glutamate via pyrroline-5-carboxylate, aids in wound healing and provides nutrition [26]. 

## 4. Discussion

Halari donkey milk is observed as a nutritive source exhibiting overall good physicochemical properties, consisting of a decent amount of minerals, vitamins, and amino acids. The nutritional composition of Halari donkey milk recorded in this current study demonstrated that the essential components of Halari donkey milk, particularly lactose, were similar to those of human milk. When compared to the milk of ruminants (cows, sheep, and goats), equine and human milk showed a more similar gross composition in terms of ash, lactose, and proteins, with the exception of the lower lipid content reported for donkey milk, according to the findings of past research on milk composition [5,27,28]. We discovered a mean fat content of 0.86 ± 0.04% in the latter, which is around four times lower than in human milk (3.5–4.0%) and in cow milk (3.5–3.9%), while showing a similarity to the fat content reported in mare milk (0.5–2.0%) [5]. Fat content of donkey milk varies with the change in breed. When the data of this current study was compared with previously reported literature of different breeds of donkeys, it was recorded that the fat content in Halari donkey milk was higher than reported in Sardinian donkey milk (0.7%) [8], 0.53% in Amiata donkey milk [9], and 0.42–0.72% in Martina Franca pluriparous jennies milk [29], and was in correlation with that of Arcadian donkey milk (0.7–1.3%) [7]. The lower fat concentration gives Halari donkey milk a light, fresh, and clean flavour, which also inhibits the development of an aftertaste. Rare sensory assessments of donkey milk have revealed that it has a thin texture, a mildly sweet agreeable flavour, no lingering aftertaste, a grassy aroma, and a flavour reminiscent of almonds [29,30]. Overall, Halari donkey milk may be especially helpful to some consumer groups, such the elderly, due to its low-fat level. Donkey milk’s low-fat content and quality in terms of the ratio of saturated to unsaturated fatty acids were found to be beneficial for treating and preventing atherosclerosis [31] obesity [32] and is beneficial for people with cardiovascular diseases [33].

The lactose percentage in current research was similar to the findings previously reported for Indian small grey breed donkey milk, i.e., 6.03–6.96% [13] and 6.5% reported by Murgia et al. (2016) [8]. Massouras et al. (2017) also observed similar results of 5.8–6.1% in pluriparous Arcadian donkey milk analysed throughout the lactation stage [7]. The high concentration of lactose content in Halari donkey milk is regarded to be similar to that in human milk, which makes it favourable for the consumption of children [2]. The high lactose content is responsible for the milk’s palatability and aids in the intestinal absorption of calcium, which is necessary for infant bone mineralization [34]. The total carbohydrate content and lactose content in Halari donkey milk showed differences as an elevated level of carbohydrates was observed in this current study. The primary carbohydrate in donkey milk is lactose. Lactose is a disaccharide which, during hydrolysis, produces one galactose and one glucose molecule, and is a significant source of energy. It is the primary carbohydrate in donkey milk, but some oligosaccharides (part of carbohydrates) have also been reported in donkey milk, in addition to lactose. Lictra et al. (2019), in their research, found that donkey milk contains seven distinct sialylated milk oligosaccharides [35]. These oligosaccharides are a complex class of carbohydrates that do not directly contribute to the nutritional needs of the offspring but, instead, operate as a bioactive component in several physiological and defence processes [36,37]. The presence of oligosaccharides in our milk samples can be hypothesised to be the cause of the elevated total carbohydrate levels in our study. The carbohydrate concentration in Halari donkey milk is comparable to that of mare (5.8–7.0%) and human milk (6.3–7.0%), and higher to cow milk (4.4–4.9%) and other ruminants [5]. 

In this study, the casein content found in Halari donkey milk was 0.84 ± 0.01%, which was within the range (0.64–1.03%) reported by Guo et al. (2007) [5]. Similar, rather lower values of casein 0.56 g/100 g were reported in the study of Malacarne et al. (2019) in Ragusano donkey milk samples [11]. The result in our study lies within the range (0.14–1.14 g/100 g) as reported in this study in Ragusano donkey milk samples. The low amount of casein content in donkey milk owes to its low allergenicity [38]. Fantuz et al. (2020) also reported similar values of casein content (0.62%) in donkey milk [39]. Casein content in Halari donkey milk was observed to be higher than that in human milk (0.32–0.42 g/100 g) [5]. In terms of total solids and solids not fat, Halari donkey milk was characterized to have higher values than reported by Massouras et al. (2017) in their study, which focussed on the compositional study of donkey milk throughout the lactation stage [7]. The findings of this current study were higher than the reported values in the Balkan donkey breed and Indian small grey breed in terms of its total solids and solid not fat [13,14]. The results were supported by the findings of Guo et al., 2007, reporting total solids in donkey milk in the range of 8.8–11.7 g/100 g, which were lower to human milk (10.9–13.0 g/100 g) [5]. The titratable acidity of Halari donkey milk was similar to that of human milk (0.02–0.07%) and lower than cow milk (0.166%) [40]. The energy calculated in Halari donkey milk was similar to the findings of 40.5–49.0 kcal/100 g reported by Guo et al. (2007) [5] and 38.4–43.0 kcal/100 g by Claeys et al. (2014) [41]. The water activity of Halari donkey milk was similar to that of Indian small grey breed donkey milk (0.980–989) [13]. The protein content in Halari donkey milk was found to be slightly higher than that of human milk (1.5–1.8%) and mare milk (1.5–2.8%), whereas it was lower than that of cow, sheep, goat, and buffalo milk [5,41].Comparing the protein content in Halari donkey milk with different donkey breeds, it was observed that the protein content analysed in this current study was higher to that of reported values: 1.5% in Sardinian donkey milk [8], 1.5–1.7% in Arcadian donkey milk [7], 1.63% in Amiata donkey milk [9], and 1.40–1.92% in the Balkan donkey [14].

The milk’s minerals serve a crucial role in growth, skeletal structure development, and numerous other biological processes. The findings of mineral content in Halari donkey milk differed slightly from those of earlier research on donkey milk [7,10]. The average ash content in Halari donkey milk was slightly higher than human (0.2–0.3%) and similar to mare milk (0.3–0.5%) but lower to cow milk (0.7–0.8%) [5]. These results demonstrates that donkey milk is superior to ruminant milk samples as a breast milk substitute for infants due to its low ash concentration and lower protein content, which are better suited to infants with limited renal capacity. Therefore, the renal burden was reported to be nearly identical in breastfed and donkey-fed newborns [42]. In comparison to other dairy animals among the macro elements, the calcium and magnesium concentration in Halari donkey milk resulted to be higher than human milk and similar to mare milk, whereas it was lower than sheep, buffalo, goat, and cow milk [41]. Along with calcium, phosphorus was among the highest minerals in donkey milk as it is present in association with calcium in milk. Calcium in milk is either dissolved as free ions or ion pairs in milk serum or disseminated as calcium phosphate nanoclusters coupled to caseins. In human milk, approximately 15% of Ca is linked with the casein pellet but in cow milk, 65% of Ca is connected with casein [43]. Fantuz et al. (2020) investigated the distribution of macro elements in the major fractions of donkey milk, including whole milk, fat, casein, whey, and aqueous fraction [39]. In this investigation, the majority of Ca was shown to be associated with casein, whereas the remainder was predominantly found in the aqueous phase. Ca was coupled with casein at a rate of 62.9%, whey proteins at a rate of 4.78%, and the aqueous phase at a rate of 32.3% [39]. Halari donkey milk macro minerals concentrations were in accordance with the findings of Massouras et al. (2017). In comparison to this study, calcium in Halari donkey milk was higher than the reported 53.5–94.7 mg/100 mL [7]. Similar results were obtained while comparing with the data reported by Fantuz and team. The calcium concentration was lower in Halari donkey milk, along with lower potassium concentration (74.6 mg/100 mL) reported by Fantuz et al. (2012) in their study [10]. Potassium and sodium findings were found to be in accordance with human and mare milk, while it was lower than goat, sheep, buffalo, and cow milk. The concentration of zinc present in Halari donkey milk was higher than the reported values for different mammals [41]. The discrepancies between the present results and those for donkey milk reported the literature may be because of variations in breed, lactation stage, milk protein concentration, sample size, and analytical method accuracy.

The results of vitamin concentration in Halari donkey milk were comparable to human milk, except for the concentration of vitamin B1 (thiamine) and B7 (biotin), which were higher than those in human milk [41]. The concentration of thiamine was similar to the data reported for donkey milk as 0.021–0.060 mg/100 mL by Medhammar et al. (2012) [44] and lower to the concentration (0.09–0.1 mg/100 g) reported in small grey donkey milk [13]. In this study, we discovered that the amount of riboflavin (vitamin B2) in Halari donkey’s milk was slightly higher than that of human milk but significantly lower than the levels of riboflavin in bovine milk [41]. Niacin (vitamin B3), commonly known as nicotinic acid, is a vitamin with lipid-lowering properties that can decrease triglycerides and blood cholesterol. Niacin content in Halari donkey milk was lower than in human milk and bovine milk [45]. Vitamin B5 (pantothenic acid) in Halari donkey milk was slightly lower to cow (0.260–0.490 mg/100 mL), was in corelation with mare (0.227–0.300 mg/100 mL), and buffalo milk (0.150–0.370 mg/100 mL). Vitamin B6 was lower in concentration than mare (0.030 mg/100 mL), cow milk (0.030–0.070 mg/100 mL) and buffalo milk (0.025–0.330 mg/100 mL) [41]. Vitamin B6 (pyridoxin) in Halari donkey milk was similar to the concentration present in human milk (0.011–0.014 mg/100 mL). Vitamin B6 was just established for the first time, by Vincenzetti et al. (2020) in their study, that donkey milk contains vitamin B6 in addition to other B-complex vitamins [46]. Vitamin B12 (cyanocobalamin) was not detected in Halari donkey milk. The absence of vitamin B12 in this current study is supported by the conclusions reported from an analysis of donkey milk taken from Indian tiny grey donkeys and Amiata breeds [13,46]. Given that vitamin B12 is created by the microbes of the digestive tract, the lack of this vitamin in donkey milk as opposed to cow’s milk may be explained by the differences in the digestive systems of these two animals [46]. The detailed research of vitamin B12 metabolism in donkeys will be fascinating. Amino acids are essential because they serve as building blocks for neurotransmitters and proteins, as transport molecules, aiding in cell signalling. They are also more quickly absorbed by the gut when they are in their free form. Essential amino acids such as leucine, lysine, methionine, and phenylalanine observed in Halari donkey milk had lower concentrations than the values previously depicted by Guo et al., 2007, for donkey, mare, and cow milk [5]. Lysine was present in higher concentrations in Halari donkey milk as compared to the data reported in previous literature for donkey milk, and was similar to those of mare milk, whereas it had lower concentration than that of cow milk [5]. The results of this present study in context of non-essential amino acids were lower than cow and mare milk, while concentrations of histidine and arginine were in correspondence with the findings reported for donkey milk by Guo et al. (2007) [5].

## 5. Conclusions

Donkey milk exhibits peculiar nutritional qualities similar to those found in human milk. Therefore, it can be considered as an excellent substitute. In the last decade, extensive research has been undertaken on the milk of several donkey breeds due to its distinctive qualities. This current study reveals that Halari breed milk has good nutritional value and is nearly identical in composition to human milk. The high lactose content in Halari donkey milk makes it palatable and acceptable in terms of its taste attribute. Halari donkey milk offers an excellent opportunity to be employed in the production of low-fat dairy products due to its lower fat content. The gross vitamin, mineral, and amino acid composition of Halari donkey milk are consistent with those reported for other breeds with slight differences. The low microbial load in Halari donkey milk makes it microbiologically safe and contributes to its longer shelf life. More extensive research can be conducted for Halari donkey milk to provide more conclusive insights about its nutritional qualities and possible derivatives.

## Figures and Tables

**Table 1:** Descriptive statistics of physicochemical properties of Halari donkey milk.

Composition	Range	Mean Values	SD
Total solids (%)	9.54–9.69	9.61	0.08
SNF (%)	8.70–8.96	8.81	0.14
Fat (%)	0.81–0.89	0.86	0.04
Protein (%)	2.00–2.05	2.03	0.03
Carbohydrates (%)	6.10–6.45	6.23	0.19
Lactose (%)	5.65–5.93	5.75	0.15
Ash (%)	0.46–0.56	0.51	0.05
Casein (g/100 g)	0.83–0.85	0.84	0.01
Titratable acidity	0.03–0.04	0.03	0.01
Energy (kcal/100 mL)	40.03–40.57	40.39	0.31
Water activity (a_w_)	0.973–0.975	0.974	0.001
Total plate count (cfu m/L)	3.5 × 10^4^–3.9 × 10^4^	3.73 × 10^4^	0.21
Yeast and mould count (cfu m/L)	2.9 × 10^4^–3.2 × 10^3^	3.03 × 10^3^	0.15

SNF = solids not fat; SD = standard deviation.

**Table 2:** Minerals in Halari donkey milk.

Minerals (mg/100 mL)	Range	Mean Values	SD
Magnesium	6.20–7.90	7.27	0.76
Sodium	12.65–14.89	13.84	0.92
Calcium	60–62.24	61.25	0.93
Potassium	46.55–48.24	47.39	0.69
Phosphorus	32.10–33.96	32.99	0.76
Zinc	1.78–1.89	1.83	0.05

SD = standard deviation.

**Table 3:** Vitamin concentration in Halari donkey milk.

Vitamins	Range	Mean Values	SD
Vitamin B1, Thiamine (mg/100 mL)	0.023–0.033	0.028	0.01
Vitamin B2, Riboflavin (mg/100 mL)	0.006–0.007	0.006	0.001
Vitamin B3, Niacin (mg/100 mL)	0.135–0.173	0.153	0.02
Vitamin B5, Pantothenic acid (mg/100 mL)	0.206–0.238	0.224	0.02
Vitamin B6, Pyridoxine (mg/100 mL)	0.010–0.014	0.012	0.02
Vitamin B7, Biotin (mcg/100 mL)	6.808–6.957	6.659	0.39

SD = Standard Deviation.

**Table 4:** Amino acids concentration in Halari donkey milk.

Amino Acid (g/100 g)	Range	Mean Values	SD
Essential amino acids			
Isoleucine	0.07–0.10	0.087	0.02
Leucine	0.246–0.358	0.291	0.06
Lysine	0.164–0.185	0.183	0.02
Methionine	0.011–0.016	0.014	0.003
Phenylalanine	0.01–0.03	0.02	0.01
Valine	0.70–0.90	0.80	0.10
Non-essential amino acids			
Arginine	0.04–0.09	0.070	0.03
Histidine	0.02–0.04	0.030	0.01
Proline	0.104–0.108	0.107	0.00
Serine	0.051–0.055	0.055	0.00
Alanine	0.011–0.014	0.012	0.002
Glycine	0.01–0.02	0.013	0.01
Aspartic acid	0.0051–0.0054	0.005	0.0002
Glutamic acid	0.380–0.447	0.412	0.03

SD = standard deviation.

## Data Availability

Data are contained within the article.
